# BRIDGING LONG-TERM CARE AND ELDER ABUSE RESEARCH IN A MENTOR–MENTEE COMMUNITY OF PRACTICE

**DOI:** 10.1093/geroni/igae098.0265

**Published:** 2024-12-31

**Authors:** Amy Roberts, E-Shien Chang, Tina Kilaberia, Anthony Rosen, Gary Epstein-Lubow, Mark Lachs

**Affiliations:** Miami University, Oxford, Ohio, United States; Weill Cornell Medical College, New York, New York, United States; New York University, New York, New York, United States; New York Presbyterian Hospital/Weill Cornell Medical Center, Weill Cornell Medicine, New York, New York, United States; Education Development Center, Waltham, Massachusetts, United States; Weill Cornell Medicine, New York, New York, United States

## Abstract

The National Collaboratory to Address Elder Mistreatment (NCAEM) Mentorship Program serves as an emerging hub to learn from thought leaders, build partnerships, and forge collaborations in elder mistreatment research, policy, and advocacy efforts. This one-year program brings together clinicians, service providers, researchers, policymakers, and advocates from many disciplines who share a common goal of eradicating elder mistreatment. Our session will focus on the critical role of the NCAEM in catalyzing interdisciplinary elder abuse research in long-term care settings, including nursing homes and assisted living. We will first identify approaches, mechanisms, and lessons learned in building successful and sustainable mentoring experiences through the support of the NCAEM. Perspectives from both mentees and mentors will be highlighted to reflect the range of experiences, such as mentor-mentee meetings, peer-to-peer mentoring within and across mentee cohorts, and national convenings. To demonstrate the rich collaborative experience of the NCAEM community of practice, we will then present three completed and ongoing impact-driven research projects on resident-to-resident mistreatment in long-term care settings, including: (1) characterizing racial/ethnic conflicts among residents in long-term care, (2) recognizing resident-to-resident mistreatment as a key barrier to an age-friendly environment, and (3) identifying the training and program needs for directors of social services in nursing homes regarding resident-to-resident mistreatment. Lastly, we will discuss learning using the ongoing experience of the NCAEM as a model research network model to advance ongoing work, generate new areas of inquiry, and grow interest in a focused gerontology topic.

